# Bacterial communities associated with the surface of fresh sweet pepper (*Capsicum annuum*) and their potential as biocontrol

**DOI:** 10.1038/s41598-020-65587-9

**Published:** 2020-05-22

**Authors:** Tshifhiwa Paris Mamphogoro, Martin Makgose Maboko, Olubukola Oluranti Babalola, Olayinka Ayobami Aiyegoro

**Affiliations:** 1 Gastro-Intestinal Microbiology and Biotechnology Unit, Agricultural Research Council-Animal Production, Private Bag X02, Irene, 0062 Pretoria South Africa; 2 Food Security and Safety Niche Area, Faculty of Natural and Agricultural Sciences, North-West University, Private Bag X2046, Mmabatho 2735, South Africa; 3 Crop Science Unit, Agricultural Research Council- Vegetable and Ornamental Plants, Private Bag X293, Roodeplaat 0001, Pretoria South Africa

**Keywords:** Biotechnology, Computational biology and bioinformatics, Microbiology, Molecular biology

## Abstract

Fresh produce vegetables are colonized by different bacterial species, some of which are antagonistic to microbes that cause postharvest losses. However, no comprehensive assessment of the diversity and composition of bacteria inhabiting surfaces of fresh pepper plants grown under different conditions has been conducted. In this study, 16S RNA amplicon sequencing was used to reveal bacterial communities inhabiting the surfaces of red and green pepper (fungicides-treated and non-fungicides-treated) grown under hydroponic and open field conditions. Results revealed that pepper fruit surfaces were dominated by bacterial phylum *Proteobacteria*, *Firmicutes*, *Actinobacteria*, and, *Bacteroidetes*. The majority of the bacterial operation taxonomic units (97% similarity cut-off) were shared between the two habitats, two treatments, and the two pepper types. Phenotypic predictions (at phylum level) detected a high abundance of potentially pathogenic, biofilm-forming, and stress-tolerant bacteria on samples grown on open soils than those from hydroponic systems. Furthermore, bacterial species of genera mostly classified as fungal antagonists including; *Acinetobacter*, *Agrobacterium*, and *Burkholderia* were the most abundant on the surfaces. These results suggest that peppers accommodate substantially different bacterial communities with antagonistic activities on their surfaces, independent of employed agronomic strategies and that the beneficial bacterial strains maybe more important for peppers established on open fields, which seems to be more vulnerable to abiotic and biotic stresses.

## Introduction

Fresh products such as apples, grapes, peaches, and tomatoes are known to harbour diverse bacterial populations^1–3^. Plant species, geographic location, climatic conditions, ripening stage and application of agrochemicals, are some of the factors that determine distribution of microorganisms on the surface of these products^4^. Bacterial species that colonise fruit surfaces (epiphytes) are introduced from the soil to the host plants by insects, air currents and other animal species^5–7^. Among these microorganisms, some are beneficial to plants, for example, several *Sphingomonas* strains induce resistance to Fusarium head blight caused by *Fusarium culmorum* in the host plant^8^; while others are phytopathogens (e.g., *Phoma* and *Pantoea*) known to cause economic loses^1,9^. Therefore, understanding the diversity and ecology of epiphytic bacteria may be important to develop new biocontrol agents^10^.

Previously, the identities of the members of microbial communities were established using culture-dependent methods^11^. However, these methods are known to underestimate microbial diversity, as only 0.1–8.4% of environmental bacteria are considered cultivable^12,13^. Data gathered using these methods only provide limited information on the vast majority of microbes present in a given sample. Nowadays, the diversity of bacterial communities is usually assessed by culture-independent techniques that include the analysis of the 16S rRNA gene fragments^14^. Such methods have allowed for instance the investigation of the microbial diversity of tomato, grape, peach and apple fruits^15,16^. However, information on bacterial communities associated with the surface of fresh sweet pepper fruits is still limited, despite this being vital in identifying microbes that can antagonize the effects of pathogenic strains which may contribute to postharvest loses.

The primary goal of this study was to investigate, using 16S rRNA gene Illumina amplicon sequencing, how the effect of growing conditions (hydroponic system versus direct sowing), inorganic pesticides treatment (i.e., application of a fungicide) and maturity status (green versus red), could influence the structure and composition of bacterial communities on the surfaces of fresh pepper fruits. Additionally, we aimed to predict the phenotypic changes in the microbiota of pepper samples and also, to identify bacterial taxa with potential to minimize postharvest losses of peppers.

We hypothesized that, regardless of agronomic management approaches, pepper fruits can accommodate antagonistic bacteria on its surfaces that can potentially minimize damage that maybe induced by its potential pathogens, and that some of these antagonists, may contribute in reduction of post-harvest loses.

## Results and discussion

Analysing the bacterial communities associated with the surface of *Capsicum annuum* fruits, we obtained 1,586,400 bacterial high-quality reads, which resulted in 1,137 OTUs (97% cut-off). The majority of bacterial OTUs were shared between the habitats, treatments and pepper sample types (56.4%, 58.9% and 59.4%, respectively) (Supplementary Fig. S1). Microbial diversity (Supplementary Fig. S2) tended to be higher in the fungicide-treated compared to fungicide-untreated samples, in open field compared to the hydroponic system samples, and in the green compared to the red samples, although they did not differ significantly (*P* > 0.05). This implies that microbial diversity on the surfaces of peppers is not affected by growth stage, growing system and treatment with fungicides. For habitats, diversity results were as expected, as it is well known that the organic matter in the soil is an important source of nutrients for microorganisms and contains higher levels of fungal and bacterial propagules than hydroponic systems^17^. The diversity in treatments is in agreement with a study by Schaeffer, *et al*.^18^, which showed fungicides application on nectar have no observable effect on bacterial OTU richness or community compositions. Furthermore, higher bacterial populations observed on the immature (green) fruit surfaces compared to the mature samples corroborated with findings by Palumbo, *et al*.^19^, who found greatest bacterial diversity on early summer mature almond fruits than on the late summer mature almonds. The possible explanation for this observation is that the intact hulls during the immature growing stage of fruits will still be metabolically active and therefore, could be ideal sources of carbon and water for microbial survival.

**Figure 1:**
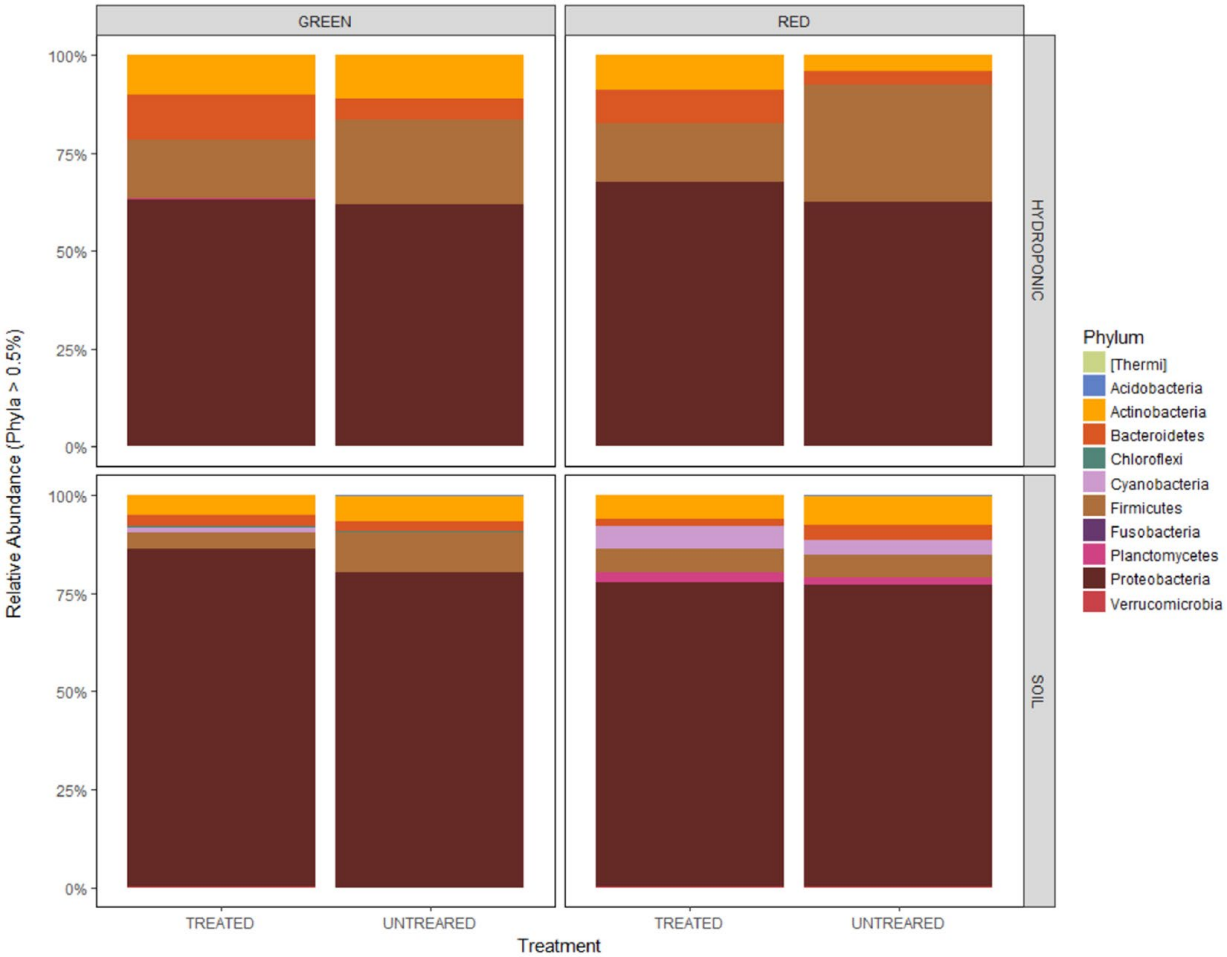
Mean relative abundances of taxa (phylum); (**a**) between hydroponic and soil habitats samples, (**b**) green and red samples, (**c**) treated and untreated samples. The abundance of each taxon calculated as the percentage of sequences per location for a given microbial group.

A total of 17 distinct bacterial phyla were detected across all 80 samples. The most abundant sequences in all the 80 samples were affiliated with the phylum *Proteobacteria* (71%), followed by *Firmicutes* (13%), *Actinobacteria* (7%) and *Bacteroidetes* (5%) (Fig. 1). Other phyla were also represented, although in lower proportions. There were significant differences in *Proteobacteria* abundance between the two habitats, with the phylum being more abundant in open soil as compared to the hydroponic habitat (Kruskal-Wallis: *P* < 0.001), but non-significant differences were observed between the two treatment groups (Kruskal-Wallis: *P* = 0.55) and the two pepper sample types (Kruskal-Wallis: *P* = 0.53). For *Firmicutes*, significant differences in abundance were shown between the habitats (Kruskal-Wallis: *P* < 0.001) and treatments (Kruskal-Wallis: *P* = 0.03), but non-significant differences in abundance were noted between the pepper sample types (Kruskal-Wallis: *P* = 0.71). Moreover, abundance of *Actinobacteria* did not differ between habitats (Kruskal-Wallis: *P* = 0.34), treatments (Kruskal-Wallis: *P* = 0.68) and pepper sample types (Kruskal-Wallis: *P* = 0.34). Additionally, significant differences in abundance were shown between habitats (Kruskal-Wallis: *P* < 0.001) and pepper sample types (Kruskal-Wallis: *P* = 0.03) for the *Bacteroidetes*, while abundances between treatment groups were not significant (Kruskal-Wallis: *P* = 0.06). The trend in abundance of *Proteobacteria* in habitats could be explained by the fact that this phylum is commonly identified as being copiotrophic (i.e., they thrive in conditions of elevated carbon availability and exhibit relatively rapid growth rates and compete successfully when organic resources are abundant), possibly because they associate with nematodes soil layers where organic matter, plant roots, and other resources are more abundant^20,21^. *Proteobacteria*, *Firmicutes*, *Actinobacteria* and *Bacteroidetes* have been shown to be widely represented on the surfaces of fruits of other plants such as grape^22^. They represent various taxonomic groups and different ecological statuses, such as antagonist, symbionts (especially, endophytes) and saprophytes^23^. Their dominance on fruit surfaces could be attributed to the fruit’s ability to use a wide variety of carbon sources such as carbohydrates, amino acids, and lipids, which could help resist different environmental changes that occur during fruit development^24,25^.

**Figure 2:**
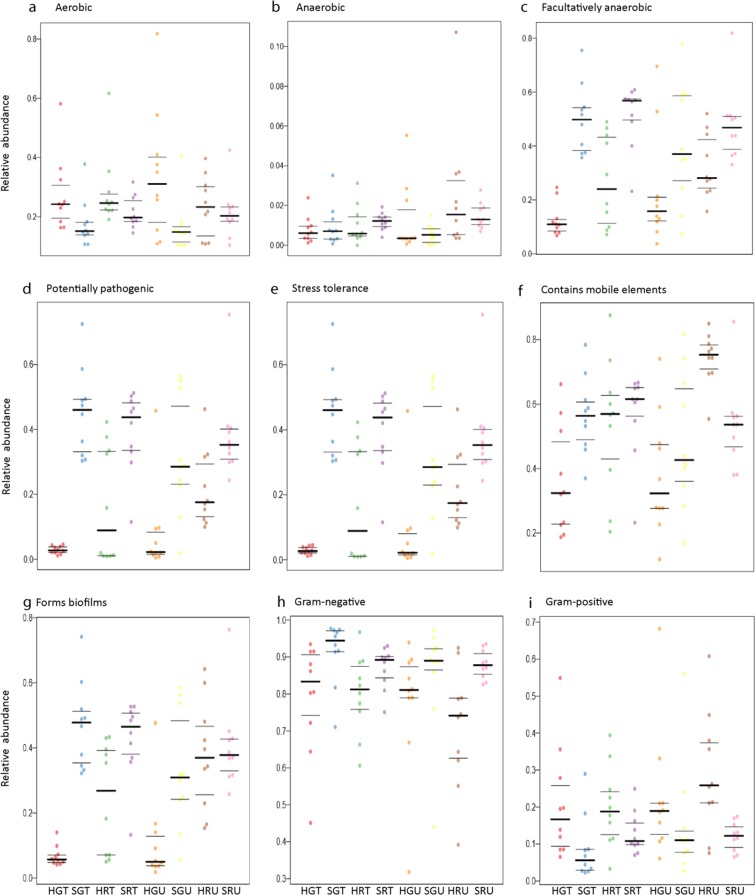
Phenotypic prediction based on BugBase analysis. Prediction of phenotypic differences from 16S rRNA sequence data associated with aerobic, potentially pathogenic, stress tolerance, mobile element, biofilms formation, Gram-negative bacteria and Gram- positive bacteria from sample between hydroponic and soil treated and untreated pepper samples.

Potential prediction of phenotypic functions of bacterial communities (at phylum level) on the surfaces of the different pepper samples detected nine potential microbial phenotypes including; aerobic, anaerobic, facultative anaerobic, mobile elements carriers, biofilm forming, Gram-negative, Gram-positive and pathogens (Fig. 2; Supplementary Table S1). In general, aerobic bacteria were more abundant on fungicide-treated compared to untreated samples and this was opposite for anaerobic bacterial populations. This could suggest that the rise in abundance of aerobic bacteria is associated with the capability of degrading fungicides by these bacteria as described by Megadi *et al*.^26^. On another note, potentially pathogenic bacteria showed to be more overrepresented on surfaces of both, immature (green) and mature (red) peppers grown on open field (both fungicide-treated and untreated). This was not the case with peppers grown under the hydroponic system, and this clearly demonstrates that, growing peppers using the hydroponic system maybe an effective agronomic management strategy in comparting yield constraining effects of microbial pathogens of peppers. Therefore, using the hydroponics technology, crops can be grown with minimal negative effects on ecosystems and biodiversity, which are usually profound in cropping systems dependent on synthetic pesticides for control of pests and diseases^27–29^. Although the initial investment of hydroponic systems in huge^30^, it may tend to be a cheaper method of growing high-value, horticultural crops such as peppers, since production costs will be minimized by reduction in pesticide requirements, which are generally very expensive^31,32^. Hydroponic systems have been adopted in production of some high value crops such as tomato and lettuce^33^ and in seedling production in nurseries^33,34^.

Additionally, it is not surprising that stress tolerance functions were predicted to be more abundant on surfaces of peppers grown on open field than those grown on the hydroponic system. It is widely known that hydroponic systems raise plants free from most abiotic stresses (e.g., drought and nutrient stress) as well as biotic stresses (diseases and weeds). In addition, it is highly likely that the biofilm forming function, predicted to be present on surfaces of peppers grown on open field than hydroponic-produced peppers, is necessary to compart the various crop growth constraining factors known to be more common on the open fields. It is also highly probable that bacterial antagonists to deter potential pathogens could be exhibited by biofilm function. Likewise, pathogenicity can also be promoted by biofilm formation. Biofilms are defined as a collective of one or more types of microorganisms that can grow on many different surfaces^35^.

Differences in abundance of all the predicted nine phenotypic functions were significant (Supplementary Table S1), implying that the bacterial communities with these functions were affected by how pepper plants were managed, (e.g., fungicide treatment versus non-fungicide treatment or hydroponic versus open soil planting). It is also worth mentioning that most of these predicted functions are present in the bacterial phylum, *Proteobacteria* and *Firmicutes* (Fig. 1, Supplementary Fig. 2). According to our knowledge, no phenotypic functions on surface bacterial communities for fresh produce grown in hydroponic and open field soil or in other farming practices such as in organic and conventional practices have been reported before.

At the genus level, significant differences in abundance of *Microbispora*, *Sphingobium*, *Paenibacillus* and *Lactococcus* were noted between the treated and untreated red and green peppers produced in hydroponics. A similar observation was recorded for peppers grown in soil (Table 1). *Microbispora* species have the ability to produce phenazine-1-carboxylic acid, which is capable of controlling southern blight disease caused by the phytopathogenic fungus, *Sclerotium rolfsii*, which causes large economic losses in many crops such as *Zea mays*^36^. *Sphingobium* species were reported to produce a volatile inhibitory compound 2-methyl-1-propanol against fungus *Pseudogymnoascus destructans*^37^, while *Paenibacillus* is known to be capable of producing various plant hormones, antibiotics and hydrolytic enzymes with ability to suppress *Fusarium* wilt of cucumber (*Cucumis sativus*), which is caused by *Fusarium oxysporum* f. sp. *cucumerinum* in non-sterile, soil-less potting medium^38^. The bacterial genus *Lactococcus*, a bacteriocin producing Lactic acid bacteria (LAB), isolated from fresh fruits *Chryso-phyllum cainito* (star apple) and *Solanum stramofolium* (pea eggplant), was reported to show inhibitory activities against both Gram-positive pathogens such as *Bacillus cereus* and *Staphylococcus aureus* and the Gram-negative pathogens (e.g., *Salmonella typhimurium*)^39,40^.

**Table 1:** Comparison of bacterial genera showing significance differences; between hydroponic treated and untreated green samples, soil treated and untreated green pepper samples, hydroponic treated and hydroponic untreated red pepper samples, and between soil treated and untreated red pepper samples.

Genus	HGT		HGU		P-value
	Mean	St. error	Mean	St. error	
*Brevundimonas*	0.310	0.039	0.000	0.000	<0.001
*Chitinophaga*	0.317	0.009	0.174	0.020	<0.001
*Chryseobacterium*	0.377	1.123	0.170	0.015	<0.001
*Clostridium*	0.414	0.025	0.214	0.038	<0.001
*Microbispora*	6.929	0.042	1.577	0.014	<0.001
*Myroides*	0.660	0.083	0.000	0.000	<0.001
*Ochrobactrum*	0.088	0.001	0.000	0.000	<0.001
*Paenibacillus*	1.178	0.008	0.359	0.105	<0.001
*Pedobacter*	0.383	0.041	0.000	0.000	<0.001
*Phenylobacterium*	0.417	0.028	0.217	0.051	0.002
*Sphingobacterium*	0.721	0.010	0.213	0.017	0.026
	**SGT**		**SGU**		
*Sphingobium*	0.363	0.004	0.118	0.001	0.001
	**HRT**		**HRU**		
*Agromyces*	0.373	0.001	0.014	0.025	<0.001
*Azospirillum*	0.340	0.010	0.141	0.017	<0.001
*Bacteroides*	0.523	0.000	0.000	0.091	<0.001
*Cellvibrio*	0.384	0.000	0.000	0.045	<0.001
*Chitinophaga*	0.474	0.015	0.000	0.000	<0.001
*Clostridium*	0.355	0.000	0.000	0.016	<0.001
*Corynebacterium*	0.477	0.006	0.126	0.037	<0.001
*Exiguobacterium*	0.400	0.004	0.104	0.044	<0.001
*Geobacillus*	1.158	0.052	0.230	0.120	<0.001
*Paenibacillus*	1.645	0.008	0.642	0.097	<0.001
*Phenylobacterium*	0.370	0.012	0.149	0.039	<0.001
*Serratia*	0.358	0.000	0.000	0.603	<0.001
	**SRT**		**SRU**		
*Agromyces*	0.688	0.000	0.000	0.008	<0.001
*Clostridium*	0.488	0.004	0.015	0.056	<0.001
*Comamonas*	0.701	0.004	0.115	0.037	<0.001
*Corynebacterium*	0.950	0.008	0.133	0.242	0.002
*Geobacillus*	0.406	0.014	0.200	0.027	<0.001
*Klebsiella*	0.364	0.001	0.098	0.054	<0.001
*Lactococcus*	2.312	0.116	0.657	0.007	0.001

Average relative abundance of sequences assigned to genus (Mean) constituting 0.3% or more sequences in either of the sample, standard error of the corresponding average (St. error) and p-value (p < 0.05 significant) describing the significance of the differential abundance observed between the two sample sources.

**Figure 3:**
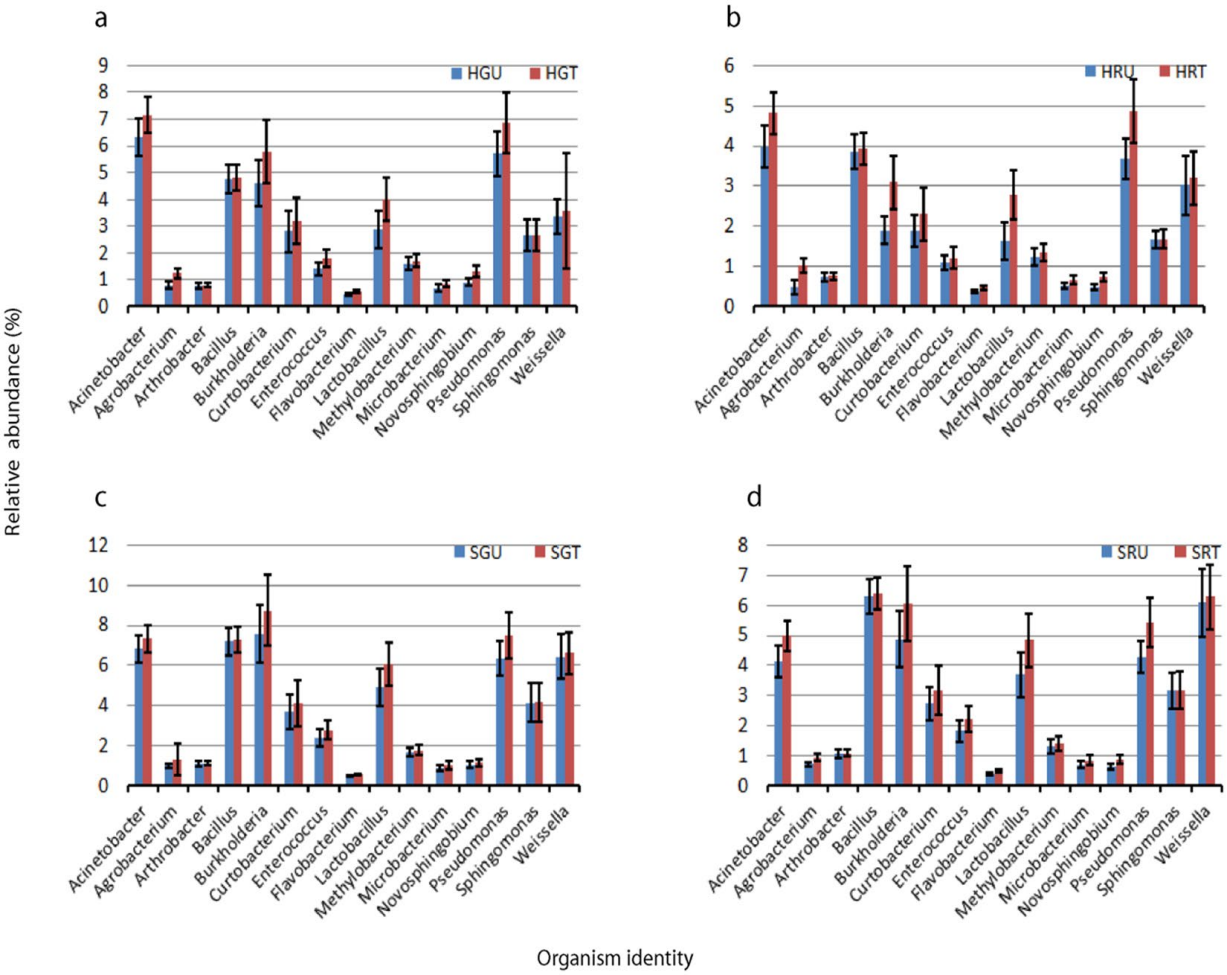
Relative proportion of bacterial antagonists (mean ≥0,3); (**a**) between hydroponic green untreated and hydroponic treated green samples, (**b**) hydroponic red untreated and hydroponic red treated samples, (**c**) soil green untreated and soil green treated samples, (**d**) soil red untreated and soil red treated samples. Error bars indicate mean ± SE.

Other well-known bacterial genera such as: *Acinetobacter, Agrobacterium, Arthrobacter, Bacillus, Burkholderia, Curtobacterium, Enterococcus, Flavobacterium, Lactobacillus, Methylobacterium, Microbacterium, Novosphingobium, Pseudomonas, Sphingomonas* and *Weissella*, were represented by the majority of sequences, but no significant differences in abundance for these genera were observed between the hydroponic-green-treated (HGT) and the hydroponic-green-untreated (HGU) samples, the soil-green-treated (SGT) and the soil-green-untreated (SGU) pepper samples, the hydroponic-red-treated (HRT) and the hydroponic-red-untreated (HRU) samples as well as between the soil-red-treated (SRT) and the soil-red-untreated (SRU) pepper samples (Fig. 3, Supplementary Table S2a,b). These genera are known to have an antagonistic action against fungal pathogens, reducing cucumber Fusarium wilt, Fusarium oxysporum, and other fungal pathogens while stimulating growth of other vegetable and fruit crops such as cucumbers and chickpeas^41–46^. These findings suggest that the abundance of these bacterial genera on surfaces of peppers are not affected by changes in growing conditions, maturity stage and pesticide treatment. Hence, these antagonists may be recommended for use in integrated pest management (IPM) programs, were both biological and chemical methods of pest control are recommended^47^. Similar results were obtained when the fruit surface bacterial communities living on apple fruits under conventional and organic management were compared, where only low abundance groups differed between the two environments^48^. A study by Telias, *et al*.^15^ also showed that these bacterial genera were highly abundant and variable on the surfaces of tomato fruits, but with no significant differences detected between the tomato fruit samples sprayed with surface water and groundwater. *Acinetobacter, Pseudomonas* and *Sphingomonas* were also identified in high abundance in the phyllosphere of some Atlantic rainforest tree species and cottonwood^15,49^, as well as on the leaves of field-grown tomatoes^50^.

In general, the abundance of all genera was consistently higher in pesticides-treated compared to pesticides-untreated pepper samples grown under both hydroponic and open field conditions. The same scenario was observed in the case of fruit maturity, where the relative abundances of all genera were higher in green compared to red sample types. For treatments, similar trends were observed in studies conducted by Johnsen, *et al*.^51^, which showed that some microbial groups are capable of using the applied pesticides as a source of energy and nutrients to multiply. For instance, benomyl insecticides have been found to stimulate *Pseudomonas* sp, which use the insecticide as a carbon source for growth^52^. Some pesticides inhibit certain groups of microorganisms and outnumber other groups by releasing them from competition. For example, a study by Hussain, *et al*.^53^ demonstrated that fungicide applications inhibited fungal activity of *Fusarium* and *Colletotrichum*, which led to a rapid flush in bacterial activity of *Bacillus, Acinetobacter* and *Rhodobacter*. Trends observed for fruit maturity could be explained by the fact that, (i) a cyclic changes are observed in temperature and water availability during fruit development in early summer and (ii) progressive desiccation of fruits during maturation in late summer causing pepper to become less susceptible to many bacterial species. These conditions are selective for few species including *Bacillus* as described by Nicholson, *et al*.^54^.

**Figure 4:**
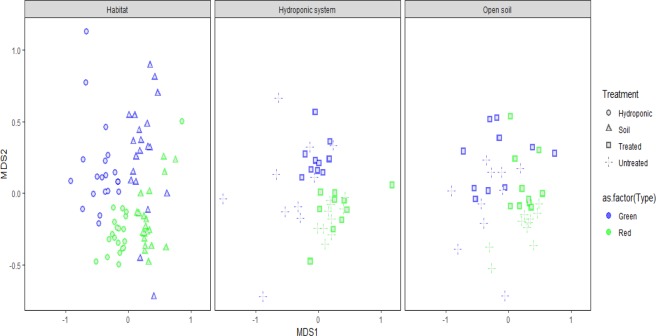
An NMDS plot showing differences in bacterial structure; (**a**) between hydroponic and soil habitat, (**b**) green and red samples under hydroponic habitat, (**c**) green and red samples under soil habitat.

Ordinating bacterial communities data using NMDS plots grouped the bacterial communities separately according to their habitats, treatements and sample type, observing distinct microbial assemblages (Fig. 4). Permutation tests revealed significant effects of habitats, pepper types and treatments on bacterial community structure and composition (i.e., PERMANOVA_Habitat_, F_1_ = 23.99, *P* < 0.001; PERMANOVA_Treatment_, F_1_ = 2.89, *P* < 0.001; and, PERMANOVA_Type_, F_1_ = 8.80, *P* < 0.001). Although differences in pepper surface bacterial community structure have been reported between organic and conventional farming practices^15^, no differences have been reported for hydroponic and open field soil. Results from the present study indicate that the bacterial community in hydroponic surface pepper is distinct from those in open field soil surface pepper, regardless of pesticides application and pepper types.

In conclusion, we have demonstrated that pepper (*Capsicum annum*) harbors diverse bacterial communities on its surfaces, independent of growing conditions, sample treatment, and sample type, which influenced their composition and abundances. Some of these bacteria are potential antagonists, which may interact with and inhibit postharvest pathogens. The likely biocontrol mechanisms by these genera involve multifaceted interactions between the host, pathogen and the antagonists which include production of extracellular cell wall degrading enzymes, competition for space, nutrients and space, production of various plant hormones, mycoparasitsm, and production of volatile organic compounds^36–38,41–44,46,55^. A large group of taxa were common across habitats, treatments and sample type. These taxa represented more than 50% bacterial phylotypes. Phenotypic predictions (at phylum level) seemed to suggest that the agronomic decision of whether to grow peppers on hydroponics or on open fields can be key as a disease control measure, as potentially pathogenic bacteria were predicted to be more abundant on samples grown on open fields than those from hydroponic systems. This finding demonstrated that hydroponic systems can be key in reducing production costs, in the long-run, as well as in preserving the integrity of ecosystems, which have for long, been under threat from high-input crop production systems that rely much of heavy inorganic pesticide and fertilizer applications. Additionally, many of the bacterial genera observed in high abundance in samples collected on plants grown under hydroponic and open field conditions are known to contain bacterial strains with plant growth promoting abilities for example *Acinetobacter*, *Arthrobacter*, *Bacillus*, *Burkholderia*, *Curtobactarium* and *Microbacterium*^56–59^ and those that act as antagonists against fungal plant pathogens^42–46^. However, a further investigation of these beneficial bacteria using culture-based approaches will help in isolating and characterizing the effects of the antagonists against bacterial pathogens of pepper. Overall, peppers can accommodate different bacterial taxa on its surfaces, some of which with beneficial functional attributes such as pathogenic microbe antagonism, but these beneficial functions will be more important for plants grown under open soils, since they will be more exposed to both, biotic and abiotic stress factors.

## Materials and methods

### Study sites and crop management

Sweet peppers were grown in the summer and autumn seasons from October 2014 to March 2015, at the Agricultural Research Council-Vegetable and Ornamental Plants (ARC-VOP), Roodeplaat, Pretoria, South Africa (25°59′S; 28°35′E and at an altitude of 1200 m above sea level). Plants were grown under both hydroponic (40% black and white shade net structure) and field conditions. The mean temperature for hydroponic growing conditions were 33 °C day/15 °C night. In the open field, temperatures of 34.5 °C day/15 °C night were recorded. The experimental design was a 2 (treatments) × 2 (growing conditions) × 2 (maturity stages) factorial, with ten replicates (n = 80). The treatments were fungicide-treated (T) and fungicide-untreated (U); the growing conditions hydroponic (H) and field (S); and the two maturity stages red (R) and green (G).

For the field experiment, seven-week-old sweet-pepper seedlings of cultivar ‘King Arthur’ of indeterminate growth habit were transplanted onto 20 cm-high ridges, with an intra-row spacing of 0.3 m and an inter-row spacing of 1.5 m. Plants were pruned to three stems and supported by horizontal twines to box the plants between horizontal twine until the height of 1.5 m. The soil was composed of a mixture of sandy, clay and loam (68%, 8% and 24%, respectively). The chemical composition of the soil (pH 7.3) was as follows: 73.1 mg.kg^−1^ phosphorus (P), 182 mg.kg^−1^ potassium (K), 978 mg.kg^−1^ calcium (Ca), 189 mg.kg^−1^ magnesium (Mg), and 51.1 mg.kg^−1^ sodium (Na). Nitrogen was applied at the rate of 180 kg.ha^−1^ and was incorporated into the soil by banding with three split applications. The first application of nitrogen was at transplanting (50%), the second four weeks after transplanting (WAT) at WAT (25%) and the last at eight WAT (25%). Superphosphate (Ca (H_2_PO_4_)_2_) and potassium sulphate (K_2_SO_4_) were applied at planting at the rate of 20 kg.ha^−1^ (10.5% P) and 40 kg.ha^−1^ (42% K), respectively. Drip irrigation supplied 550 mm water. The total rainfall received during the growing season was 40 mm.

For the hydroponic experiment, sweet-pepper seedlings as above were transplanted into 10 L plastic bags filled with sawdust as a growing medium. The drip irrigation system, with one dripper per plant, delivering 2.1 litres of nutrient solution per hour was used to fertilize the plants as described by Maboko and Du Plooy^60^. The plants were pruned to three stems at four WAT. Each stem was trellised by twisting twine around the main stem and fixing it to a stay wire 2 m above the ground surface to support the plant. Side branches were removed weekly to maintain the three-stem system.

For both hydroponic and field conditions, after two WAT plants were sprayed with the following fungicides to control powdery mildew, blight and leaf spot: COPPER-COUNT N (5 mL/L), SPOREKILL (1 mL/L), BINOMYL (50 g mL/L), BRAVO (210 mL/L) and RIDMOL (360 mL/L). Insecticides ACTARA (50 mL/L), HUNTER (40 mL/L), DIOZINON (160 mL/L), BIOMECTINE (60 mL/L), and SAVAGE (40 mL/L)) were also applied to control white flies, red spider mites and aphids.

### Sample collection and processing

Fresh, intact and healthy green and red (10 and 14 weeks after planting, respectively) sweet pepper fruit samples were aseptically collected, stored in sterile Ziploc bags and kept at 4 °C in the lab. A total of 80 samples were harvested: 10 Hydroponic-Green-Treated (HGT), 10 Hydroponic-Red-Treated (HRT), 10 Hydroponic-Green-Untreated (HGU), 10 Hydroponic-Red-Untreated (HRU), 10 Soil-Green-Treated (SGT), 10 Soil-Red-Treated (SRT), 10 Soil-Green-Untreated (SGU) and 10 Soil-Red-Untreated (SRU). Microbial biofilms on the surfaces of the pepper fruits were retrieved using sterile cotton swabs soaked in a solution containing 0.15 M NaCl and 0.1% Tween 20, as described by Paulino, *et al*.^61^. The swabs were then transferred to micro centrifuge tubes and stored at −80 °C until DNA extraction was performed.

### DNA extraction and fragment amplification and high- throughput sequencing

Genomic DNA was isolated from the 80 samples using the ZR Fungal/Bacterial DNA extraction kit (ZYMO Research, Irvine, CA, USA) according to the manufacturer’s instructions. Bacterial 16S rRNA gene amplicons were amplified using primers, 515F (5′-GTGYCAGCMGCCGCGGRA-3′) and 909 R (5′-CCCCGYCAATTCMTTTRAG-3′), targeting the V4 hypervariable region^62^. PCR was conducted in a single step using a barcoded forward primer and HotStarTaq Plus Master Mix Kit (QIAGEN, Valencia, CA). The thermocycling conditions were initial denaturation at 94 °C for 3 minutes, followed by 28 cycles of 94 °C for 30 seconds, 53 °C for 40 seconds and 72 °C for 1 minute, then a final elongation step at 72 °C for 5 minutes. PCR products were separated by electrophoresis on 2% agarose gel to observe the expected band sizes. All samples were pooled in equal proportions and purified using calibrated Ampure XP beads (Agencourt Bioscience Corporation, MA, USA). Sequencing was performed on an Illumina MiSeq platform (Illumina Inc., San Diego, CA, USA) at the Molecular Research LP next generation sequencing service (http://www.mrdnalab.com, Shallowater, TX, USA) according to the manufacture’s guidelines.

### Bioinformatics analysis

The generated 16S rRNA gene sequence data was analyzed using QIIME v1.9.1^63^. Joined sequences <200 bp long, with more than two ambiguous bases, had a quality score of <25 or more than one mismatch to the sample-specific barcode or to the primer sequences, were discarded. Chimeric sequences were discarded using USEARCH V6.1^64^. Good quality reads were clustered into operational taxonomic units (OTUs) at 97% similarity level based on the *Greengenes* reference sequence database (version 13.8) and the *de novo* OTU picking algorithm. The taxonomic affiliations of the OTUs were determined using the naive Bayesian rRNA classifier^65^ at the 80% confidence level. Singletons, chloroplast and archaea species were filtered out from the OTU table and each sample was randomly subsampled (rarefied) to 28,646 reads, which was the lowest number of sequences obtained in a given sample.

### Statistical analyses

Alpha diversity was assessed by computing richness and Shannon index using the ‘*diversity*’ function in the Vegan^66^ R package. Statistical differences were evaluated using Kruskal-Wallis tests^67^. The number of shared OTUs between communities/samples was visualized using the ‘*Venn*’ function in Gplots (cran.r-project.org/package = gplots). The OTU table was Hellinger-Transformed and the Bray-Curtis distances was used to generate a dissimilarity matrix. The structure of the microbial communities was visualized using non-metric multidimensional scaling (nMDS) plots. Permutational analysis of variance (PERMANOVA)^68^ using the ‘*Adonis*’ function in the Vegan R package was used to test for differences in bacterial composition and structure. Bugbase (http://github.com/danknights/bugbase) was used to calculate differences between both groups in terms of microbial phenotypes.

## Supplementary information

Supplementary information.

## Data Availability

The raw Illumina sequencing reads for this project have been submitted to the National Centre for Biotechnology Information Short Read Archive (SRA) database with accession no. PRJNA529905. This Targeted Locus Study (TLS) project have been deposited at DDBJ/EMBL/GenBank under the accession KDDL00000000. The version described in this paper is the first version, KDDL01000000.

## References

[1] Abdelfattah A, Wisniewski M, Droby S, Schema L (2016). Spatial and compositional variation in the fungal communities of organic and conventionally grown apple fruit at the consumer point-of-purchase. Horticulture Research.

[2] Oliveira M, Usall J, Vin˜as I, Anguera M, Gatius F (2010). Microbiological quality of fresh lettuce from organic and conventional production. Food Microbiology.

[3] Rastogi G, Sbodio A, Tech JJ, Suslow TV, Coaker GL (2012). Leaf microbiota in an agroecosystem: spatiotemporal variation in bacterial community composition on field-grown lettuce. The ISME Journal.

[4] Pinto C (2015). Wine fermentation microbiome: a landscape from different Portuguese wine appellations. Frontiers in Microbiology.

[5] Valero E, Cambon B, Schuller D, Casal M, Dequin S (2007). Biodiversity of Saccharomyces yeast strains from grape berries of wine-producing areas using starter commercial yeasts. FEMS Yeast Research.

[6] Burrows SM, Elbert W, Lawrence MG, Pöschl U (2009). Bacteria in the global atmosphere—Part 1: Review and synthesis of literature data for different ecosystems. Atmospheric Chemistry and Physics.

[7] Stefanini I (2015). Role of social wasps in Saccharomyces cerevisiae ecology and evolution. Proceedings of the National Academy of Sciences of the United States of America.

[8] Liu J, Sui Y, Wisniewski M, Droby S, Liu Y (2013). Review: utilization of antagonistic yeasts to manage postharvest fungal diseases of fruit. International Journal of Food Microbiology.

[9] Lindow SE, Brandl MT (2003). Microbiology of the phyllosphere. Applied Environmental Microbiology.

[10] Müller. T, Silke. R (2014). Progress in Cultivation-Independent Phyllosphere Microbiology. FEMS Microbiology Ecology.

[11] Wagner M, Amann R, Lemmer H, Schleifer KH (1993). Probing activated-sludge with oligonucleotides specific for Proteobacteria - inadequacy of culture-dependent methods for describing microbial community structure. Applied Environmental Microbiology.

[12] Acosta-Martínez V, Dowd SE, Sun Y, Allen VG (2008). Tag-encoded pyrosequencing analysis of bacterial diversity in a single soil type as affected by management and land use. Soil Biology and Biochemistry.

[13] Aremu BR, Babalola OO (2015). Construction of specific primers for rapid detection of South African exportable vegetable macergens. International Journal of Environmental Research and Public Health.

[14] Kamutando CN (2017). Soil nutritional status and biogeography influence rhizosphere microbial communities associated with the invasive tree Acacia dealbata. Scientific reports.

[15] Telias A, White JR, Pahl DM, Ottesen AR, Walsh CS (2011). Bacterial community diversity and variation in spray water sources and the tomato fruit surface. BMC Microbiology.

[16] Leff JW, Fierer N (2013). Bacterial communities associated with the surfaces of fresh fruits and vegetables. PLoS One.

[17] Postma, J., van OS, E. & Bonanitas, P. J. M. Pathogen detection and management strategies in soilless plant growing systems. In: *Soiless culture: Theory and practice* (eds. Raviv, M. & Lieth, H. J) 425–457 (Elsevier, 2008).

[18] Schaeffer RN, Vannette RL, Brittain C, Williams NM, Fukami T (2017). Non-target effects of fungicides on nectar-inhabiting fungi of almond flowers. Environmental Microbiology Reports.

[19] Palumbo JD, Baker JL, Mahoney NE (2006). Isolation of Bacterial Antagonists of Aspergillus flavus from almonds. Microbial Ecology.

[20] Fierer N, Bradford M, Jackson. R (2007). Toward an ecological classification of soil bacteria. Ecology.

[21] Eilers KG, Lauber CL, Knight R, Fierer N (2010). Shifts in bacterial community structure associated with inputs of low molecular weight carbon compounds to soil. Soil Biology and Biochemistry.

[22] Anderson MJ (2001). A new method for non‐parametric multivariate analysis of variance. Austral Ecology.

[23] Leveau JHJ, Tech JJ (2011). Grapevine microbiomics: Bacterial diversity on grape leaves and berries revealed by High-throughput sequence analysis of 16S rRNA amplicons. Acta Horticulture.

[24] Peighamy-Ashnaei S, Sharifi-Tehrani A, Ahmadzadeh M, Behboudi K (2006). Effect of carbon and nitrogen sources on growth and biological efficacy of Pseudomonas fluorescens and Bacillus subtilis against Rhizoctonia solani, the causal agent of bean damping-off. Communications in Agricultural and Applied Biological Sciences.

[25] Kazakov AE (2009). Comparative genomics of regulation of fatty acid and branched chain amino acid utilization in Proteobacteria. Journal of Bacteriology.

[26] Megadi VB, Tallur PN, Hoskeri RS, Mulla SI, Ninnekar HZ (2010). Biodegradation of pendimethalin by Bacillus circulans. Indian Journal of Biotechnology.

[27] Römbke J, Schmelz RM, Pélosi C (2017). Effects of Organic Pesticides on Enchytraeids (Oligochaeta) in Agroecosystems: Laboratory and Higher-Tier Tests. Frontiers in Environmental Science.

[28] Tunstall-Pedoe H (2004). Pesticide pollution remains severe after clean up of a stock pile of obsolete pesticides at Vikuge, Tanzania. AMBIO A Journal of the Human Environment.

[29] Zhang L, Yan C, Guo Q, Zhang J, Ruiz-Menjivar J (2018). The impact of agricultural chemical inputs on environment: global evidence from informetrics analysis and visualization. International Journal of Low-Carbon Technologies.

[30] Sulma V, Régio G, Binotto E (2019). Economic viability for deploying hydroponic system in emerging countries: A differentiated risk adjustment proposal. Land Use Policy.

[31] Aktar W, Sengupta D, Chowdhury A (2009). Impact of pesticides use in agriculture: their benefits and hazards. Interdisciplinary Toxicology.

[32] Alavanja MCR (2009). Pesticides use and exposure extensive worldwide. Reviews on Environmental Health.

[33] Nguyen NT, McInturf SA, Mendoza-Cózatl DG (2016). Hydroponics: A Versatile System to Study Nutrient Allocation and Plant Responses to Nutrient Availability and Exposure to Toxic Elements. Journal of Visualized Experiments.

[34] Hoang NN, Kitaya Y, Shibuya T, Endo R (2019). Development of an *in vitro* hydroponic culture system for wasabi nursery plant production—Effects of nutrient concentration and supporting material on plantlet growth. Scientia Horticulturae.

[35] Davey ME, O’toole GA (2000). Microbial Biofilms: from Ecology to Molecular Genetics. Microbiology and Molecular Biology Reviews.

[36] Patil NN (2014). Potential of Microbispora sp. V2 as biocontrol agent against Sclerotium rolfsii, the causative agent of southern blight of Zea mays L (Baby corn)- *in vitro* studies. Indian Journal of Experimental Biology.

[37] Micalizzi EW, Mack JN, White GP, Avis TJ, Smith. ML (2017). Microbial inhibitors of the fungus Pseudogymnoascus destructans, the causal agent of white-nose syndrome in bats. Plos One.

[38] Garzón K, Ortega C, Tenea GN (2017). Characterization of Bacteriocin-Producing Lactic Acid Bacteria Isolated from Native Fruits of Ecuadorian Amazon. Polish Journal of Microbiology.

[39] Azhar NS, Zin NHM, Hamid THTA (2017). Lactococcus Lactis Strain A5 Producing Nisin-like Bacteriocin Active against Gram Positive and Negative Bacteria. Tropical Life Sciences Research.

[40] Larran S, Simon MR, Moreno MV, Siurana MPS, Perell A (2016). Endophytes from wheat as biocontrol agents against tan spot disease. Biological Control.

[41] Madhaiyan M (2006). Plant Growth–Promoting Methylobacterium Induces Defence Responses in Groundnut (Arachis hypogaea L.) Compared with Rot Pathogens. Current Microbiology.

[42] Wang S, Liang Y, Shen T, Yang H, Shen B (2016). Biological characteristics of Streptomyces albospinus CT205 and its biocontrol potential against cucumber Fusarium wilt. Biocontrol Science and Technology.

[43] Palmieri D, Vitullo D, De Curtis F, Lima G (2016). A microbial consortium in the rhizosphere as a new biocontrol approach against fusarium decline of chickpea. Plant and Soil.

[44] Garbeva P, Veen JA, Elsas JDV (2004). Assessment of the diversity, and antagonism towards Rhizoctonia solani AG3, of Pseudomonas species in soil from different agricultural regimes. FEMS Microbiology Ecology.

[45] Mata L, Chaves C, Rodríguez-Herrera R, Hernández-Castillo D, Aguilar C (2013). Growth inhibition of some phytopathogenic bacteria by cell-free extracts from Enterococcus sp. British Biotechnology Journal.

[46] Wachowska U, Kucharska K, Jedryczka M, Łobik N (2013). Microorganisms as biological control agents against fusarium pathogens in winter wheat. Polish Journal of Environmental Studies.

[47] Gangwar RK (2017). Role of biological control agents in integrated pest management approaches. Acta Scientific Agriculture.

[48] Ottesen AR, White JR, Skaltsas DN, Newell MJ, Walsh CS (2009). Impact of organic and conventional management on the phyllosphere microbial ecology of an apple crop. Journal of Food Protection.

[49] Lambais MR, Crowley DE, Cury JC, Bull RC, Rodrigues RR (2006). Bacterial diversity in tree canopies of the Atlantic forest. Science.

[50] Enya J (2007). Culturable leaf-associated bacteria on tomato plants and their potential as biological control agents. Microbial Ecology.

[51] Johnsen K, Jacobsen CS, Torsvik V (2001). Pesticides effects on bacterial diversity in agricultural soils—A review. Biology and Fertility of Soils.

[52] Pandey CB, Singh GB, Singh K, Singh RK (2010). Soil nitrogen and microbial biomass carbon dynamics in native forests and derived agricultural land uses in a humid tropical climate of India. Plant and Soil.

[53] Hussain S, Siddique T, Saleem M, Arshad M, Khalid A (2009). Impact of pesticides on soil microbial diversity, enzymes, and biochemical reactions. Advances in Agronomy.

[54] Nicholson WL, Munakata N, Horneck G, Melosh HJ, Setlow P (2000). Resistance of Bacillus endospores to extreme terrestrial and extra terrestrial environments. Microbiology and Molecular Biology Reviews.

[55] Mamphogoro, T. P., Babalola, O. O. & Aiyegoro, O. A. Sustainable management strategies for bacterial wilt of sweet peppers (Capsicum annuum) and other Solanaceous crops. Preprint at, 10.1111/jam.14653 (2020). 10.1111/jam.14653 32248611

[56] Huddedar SB (2002). Isolation, characterization and plasmid pUPI126 mediated indole 3 acetic acid (IAA) production in Acinetobacter strains from rhizosphere of wheat. Applied Biochemistry and Biotechnology.

[57] Raj S, Vikas V, PatelbJay K, Singh S (2019). Plant growth promoting Curtobacterium albidum strain SRV4: An agriculturally important microbe to alleviate salinity stress in paddy plants. Ecological Indicators.

[58] Singh T, Singh DK (2019). Rhizospheric Microbacterium sp. P27 Showing Potential of Lindane Degradation and Plant Growth Promoting Traits. Current Microbioogyl.

[59] Bhattacharyya PN, Jha DK (2012). “Plant growth-promoting rhizobacteria (PGPR): emergence in agriculture,”. World Journal of Microbiology and Biotechnology.

[60] Maboko MM, Du Plooy CP (2015). Effect of Plant Growth Regulators on Growth, Yield, and Quality of Sweet Pepper Plants Grown Hydroponically. HortScience.

[61] Paulino LC, Tseng CH, Strober BE, Blaser MJ (2006). Molecular Analysis of Fungal Microbiota in Samples from Healthy Human Skin and Psoriatic Lesions. Journal of Clinical Microbiology.

[62] Wang Y, Qian PY (2009). Conservative fragments in bacterial 16S rRNA genes and primer design for 16S ribosomal DNA amplicons in metagenomic studies. Plos One.

[63] Caporaso JG (2010). QIIME allows analysis of high-throughput community sequencing data. Nature Methods.

[64] Edgar RC (2010). Search and clustering orders of magnitude faster than BLAST. Bioinformatics.

[65] Wang Q, Garrity GM, Tiedj JM, Cole JR (2007). Naïve Bayesian classifier for rapid assignment of rRNA sequences in to the new bacterial taxonomy. Applied and Environmental Microbiology.

[66] Oksanen, J. *et al*. Vegan: Community Ecology Package, vR package version 2.0–2, http://cran.r-project.org/package=vegan (2007).

[67] R Development Core Team. R: A language and environment for statistical computing. R foundation for statistical computing, http://www.r-project.org/ (2014).

[68] Hollander, M. & Wolfe, D. A. Nonparametric Statistical Methods (ed. John, W.) 115–120 (Wiley & Sons, 1973).

