# Author Correction: Bacterial communities associated with the surface of fresh sweet pepper (*Capsicum annuum*) and their potential as biocontrol

**DOI:** 10.1038/s41598-021-82923-9

**Published:** 2021-02-03

**Authors:** Tshifhiwa Paris Mamphogoro, Martin Makgose Maboko, Olubukola Oluranti Babalola, Olayinka Ayobami Aiyegoro

**Affiliations:** 1 Gastro-Intestinal Microbiology and Biotechnology Unit, Agricultural Research Council-Animal Production, Irene 0062, Private Bag X02, Pretoria, South Africa; 2 Food Security and Safety Niche Area, Faculty of Natural and Agricultural Sciences, North-West University, Private Bag X2046, Mmabatho, 2735 South Africa; 3 Crop Science Unit, Agricultural Research Council- Vegetable and Ornamental Plants, Roodeplaat 0001, Private Bag X293, Pretoria, South Africa

Correction to: *Scientific Reports* 10.1038/s41598-020-65587-9, published online 22 May 2020

The original version of this Article contained errors.

In the Abstract,

“In this study, 16S RNA amplicon sequencing was used to reveal bacterial communities inhabiting the surfaces of red and green pepper (fungicides-treated and non-fungicides-treated) grown under hydroponic and open field conditions.”

now reads:

“In this study, 16S rRNA amplicon sequencing was used to reveal bacterial communities inhabiting the surfaces of red and green pepper (fungicides-treated and non-fungicides-treated) grown under hydroponic and open field conditions.”

In addition, in the Results and discussion section,

“The likely biocontrol mechanisms by these genera involve multifaceted interactions between the host, pathogen and the antagonists which include production of extracellular cell wall degrading enzymes, competition for space, nutrients and space, production of various plant hormones, mycoparasitsm, and production of volatile organic compounds^36–38,41–44,46,55^.”

now reads:

“The likely biocontrol mechanisms by these genera involve multifaceted interactions between the host, pathogen and the antagonists which include production of extracellular cell wall degrading enzymes, competition for nutrients and space, production of various plant hormones, mycoparasitsm, and production of volatile organic compounds^36–38,41–44,46,55^.”

Finally, the Supplementary Information file contained an error in the legend of Supplementary Figure S3, where.

“**Figure S3** BugBase OTU contribution phyla plots for phenotypic functions predictions; relative abundance plots of phyla predicting phenotypic functions between hydroponic and soil treated and untreated pepper samples. Contributing phyla”

now reads:

“**Figure S3** BugBase OTU contribution phyla plots for phenotypic functions predictions; relative abundance plots of phyla predicting phenotypic functions between hydroponic and soil treated and untreated pepper samples.”

These errors have now been corrected in the PDF and HTML versions of the Article, and in the accompanying Supplementary Information file.

