# Supplementary material for: Bacterial communities associated with the surface of fresh sweet pepper (Capsicum annuum) and their potential as biocontrol
Source: Sci Rep. 2020 May 22;10:8560. doi: 10.1038/s41598-020-65587-9 (PMC7244708; doi:10.1038/s41598-020-65587-9)
Supplement: Supplementary file 1 — Supplementary information. [file 41598_2020_65587_MOESM1_ESM.docx]

**Bacterial communities associated with the surface of fresh sweet pepper (*Capsicum annuum*) and their potential as biocontrol**

Tshifhiwa Paris Mamphogoro^1, 2^, Martin Makgose Maboko^3^, Olubukola Oluranti Babalola^2^, Olayinka Ayobami Aiyegoro^1*^

**Supplementary Material**

**Figure S1** Venn diagram showing the number of shared phylotypes A) between hydroponic and soil habitats, B) treated and untreated samples, and C) green and red samples communities.

**Figure S2** Diversity measures (richness, Shannon, inverse Simpson and Pielou’s evenness) of

bacterial OTUs (both 97% cut-off) A) between treated and untreated samples B) hydroponic and soil habitats and C) green and red samples.

**Figure S3** BugBase OTU contribution phyla plots for phenotypic functions predictions; relative abundance plots of phyla predicting phenotypic functions between hydroponic and soil treated and untreated pepper samples. Contributing phyla

**Table S1**. Relative abundance of nine potential phenotypes predicted by BugBase in fungicide treated and untreated samples.

**Table S2** A) and B) Bacterial genera (antagonists) in pepper fruit surface samples; between hydroponic untreated and treated green samples, and between hydroponic untreated and treated red samples.

**Figure S1**


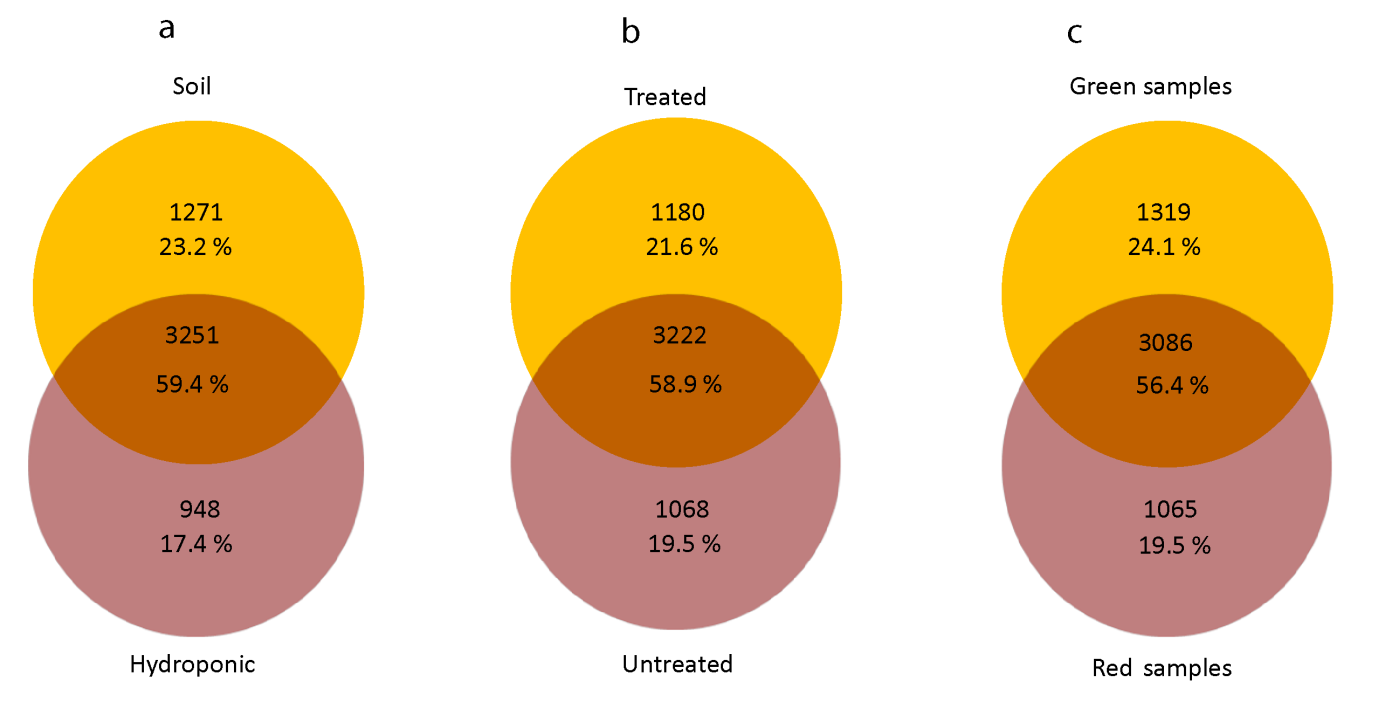


**Figure S2**

**
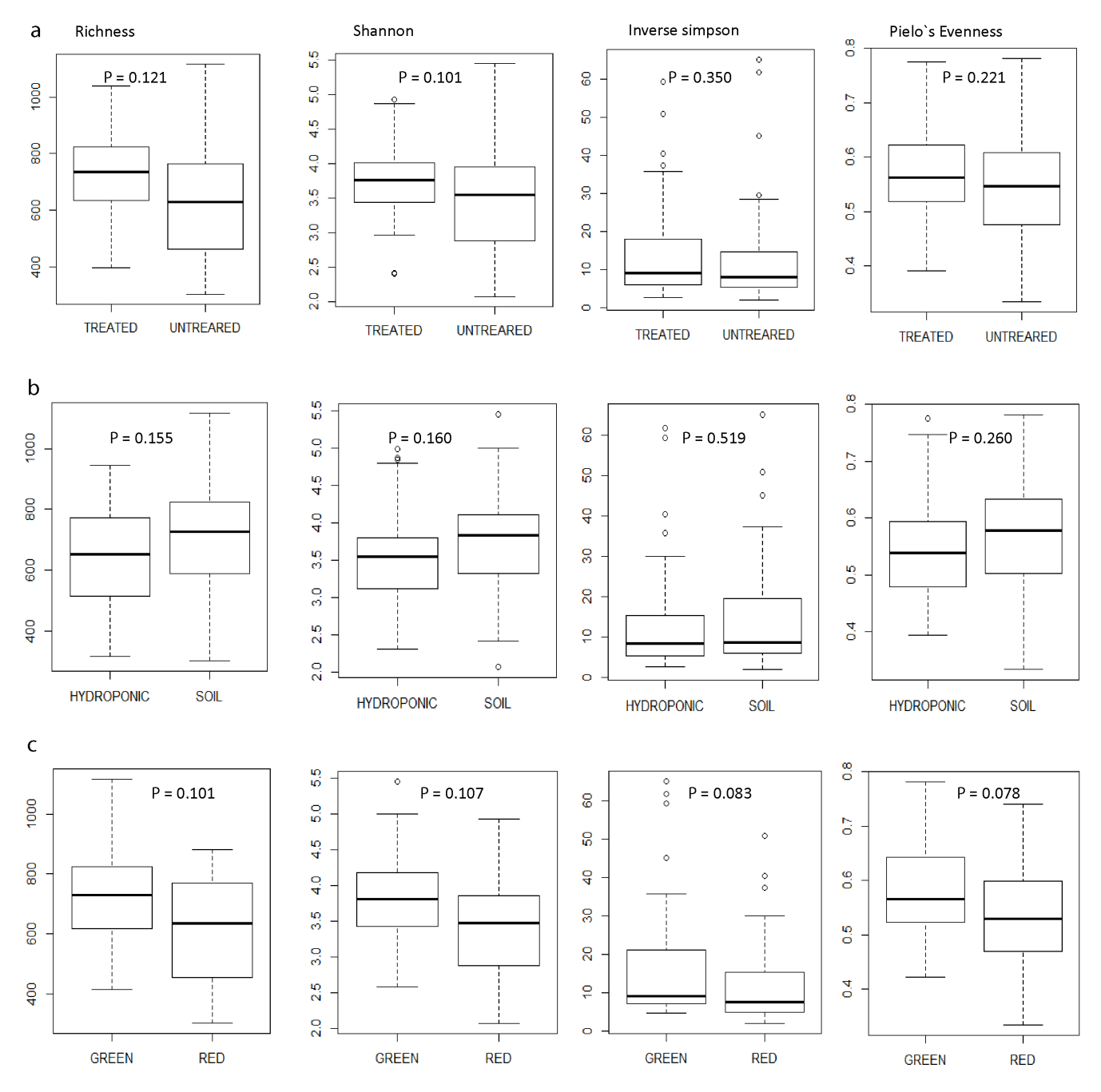
**

**Figure S3.**


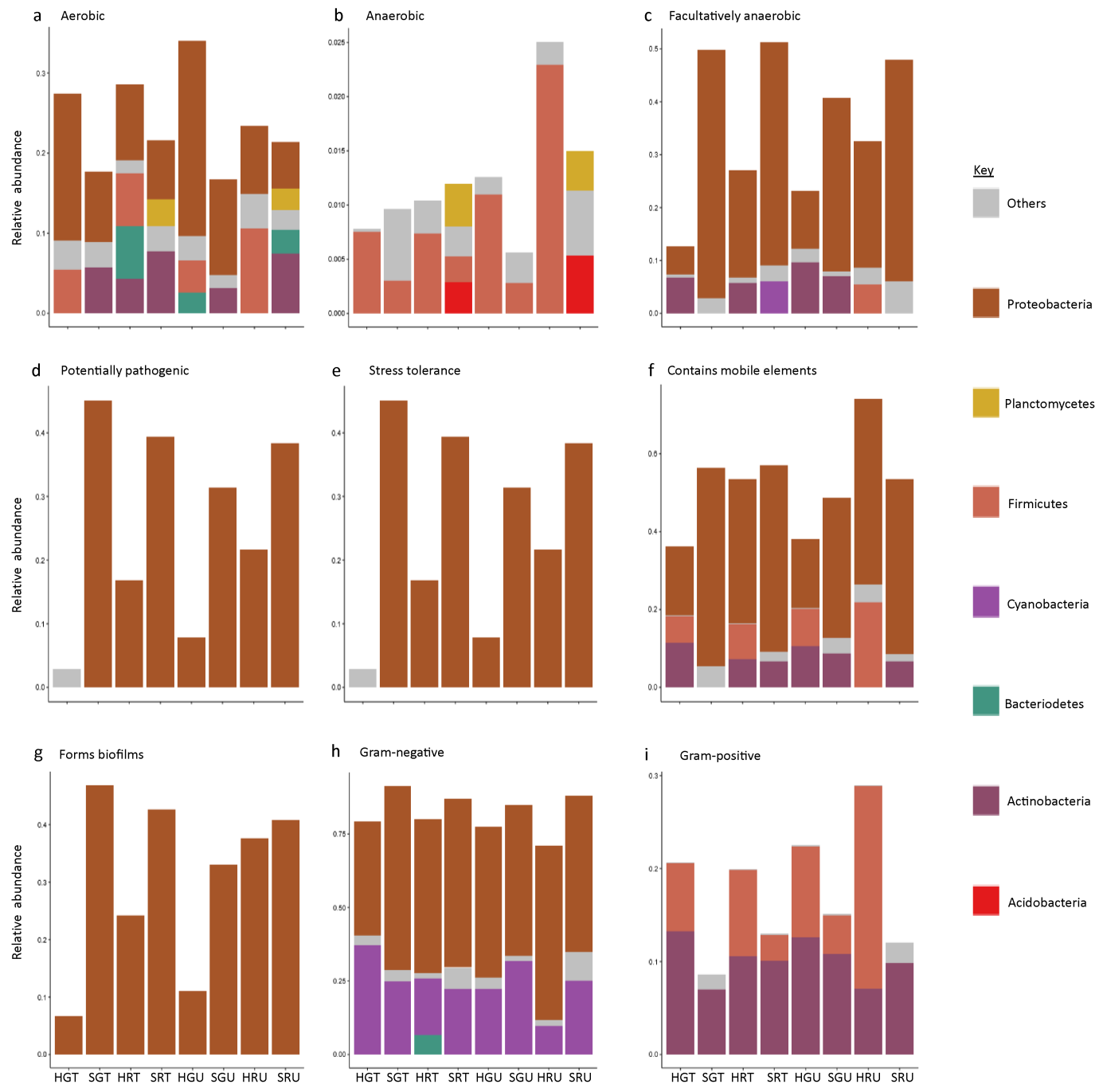


**Table S1**

Table S1. Relative abundance of nine potential phenotypes predicted by BugBase in fungicide treated and untreated samples.

| Phenotypes | HGT/PROP | SGT/PROP | HRT/PROP | SRT/PROP | HGU/PROP | SGU/PROP | HRU/PROP | SRU/PROP | P-value |
| --- | --- | --- | --- | --- | --- | --- | --- | --- | --- |
| Aerobic | 0.275 | 0.177 | 0.286 | 0.216 | 0.341 | 0.117 | 0.234 | 0.214 | 0.007 |
| Anaerobic | 0.008 | 0.009 | 0.010 | 0.012 | 0.013 | 0.025 | 0.015 | 0.006 | 0.035 |
| Contains mobile elements | 0.362 | 0.564 | 0.536 | 0.570 | 0.382 | 0.487 | 0.742 | 0.534 | <0.001 |
| Facultative anaerobe | 0.126 | 0.497 | 0.269 | 0.513 | 0.232 | 0.408 | 0.325 | 0.479 | <0.001 |
| Forms biofilms | 0.067 | 0.469 | 0.242 | 0.426 | 0.111 | 0.330 | 0.377 | 0.408 | <0.001 |
| Gram-negative | 0.793 | 0.914 | 0.800 | 0.869 | 0.775 | 0.849 | 0.710 | 0.879 | 0.003 |
| Gram-positive | 0.207 | 0.086 | 0.199 | 0.130 | 0.225 | 0.151 | 0.289 | 0.120 | 0.003 |
| Potential pathogenic | 0.029 | 0.451 | 0.168 | 0.394 | 0.079 | 0.314 | 0.217 | 0.384 | <0.001 |
| Stress tolerance | 0.029 | 0.451 | 0.168 | 0.394 | 0.079 | 0.314 | 0.217 | 0.384 | <0.001 |

HGT, hydroponic green treated; SGT, soil green treated; HRT, hydroponic red treated; SRT, soil red treated; HGU, hydroponic green untreated; SGU, soil green untreated; HRU, hydroponic red untreated; SRU, soil red untreated; PROP, Proportion.

**Table S2 (a)**

| Table S2 (a)Bacterial genera (antagonists) in pepper fruit surface samples; between hydroponic untreated and treated green samples, and between hydroponic untreated and treated red samples | | | | | | | | | | |
| --- | --- | --- | --- | --- | --- | --- | --- | --- | --- | --- |
| Genus | HGU mean | HGU SE | HGT mean | HGU SE | p-value | HRU mean | HRU SE | HRT mean | HRT SE | P-value |
| *Acinetobacter* | 6.321 | 0.689 | 7.16 | 0.684 | 0.389 | 3.981 | 0.519 | 4.819 | 0.515 | 0.254 |
| *Agrobacterium* | 0.791 | 0.123 | 1.231 | 0.194 | 0.057 | 0.479 | 0.196 | 1.023 | 0.185 | 0.045 |
| *Arthrobacter* | 0.778 | 0.096 | 0.795 | 0.091 | 0.898 | 0.74 | 0.103 | 0.756 | 0.098 | 0.911 |
| *Bacillus* | 4.745 | 0.535 | 4.826 | 0.487 | 0.421 | 3.845 | 0.434 | 3.927 | 0.387 | 0.888 |
| *Burkholderia* | 4.595 | 0.879 | 5.788 | 1.189 | 0.421 | 1.896 | 0.356 | 3.093 | 0.665 | 0.115 |
| *Curtobacterium* | 2.804 | 0.771 | 3.205 | 0.853 | 0.728 | 1.866 | 0.393 | 2.302 | 0.656 | 0.569 |
| *Enterococcus* | 1.399 | 0.253 | 1.785 | 0.325 | 0.350 | 1.089 | 0.196 | 1.212 | 0.268 | 0.712 |
| *Flavobacterium* | 0.438 | 0.04 | 0.536 | 0.051 | 0.133 | 0.368 | 0.041 | 0.465 | 0.043 | 0.105 |
| *Lactobacillus* | 2.86 | 0.698 | 4.002 | 0.828 | 0.293 | 1.641 | 0.464 | 2.783 | 0.611 | 0.139 |
| *Methylobacterium* | 1.602 | 0.229 | 1.704 | 0.229 | 0.753 | 1.226 | 0.218 | 1.329 | 0.219 | 0.739 |
| *Microbacterium* | 0.689 | 0.116 | 0.838 | 0.144 | 0.422 | 0.517 | 0.081 | 0.666 | 0.109 | 0.274 |
| *Novosphingobium* | 0.893 | 0.125 | 1.313 | 0.200 | 0.077 | 0.469 | 0.077 | 0.725 | 0.107 | 0.054 |
| *Pseudomonas* | 5.721 | 0.842 | 6.862 | 1.120 | 0.417 | 3.665 | 0.507 | 4.862 | 0.785 | 0.202 |
| *Sphingomonas* | 2.665 | 0.586 | 2.675 | 0.587 | 0.990 | 1.669 | 0.228 | 1.679 | 0.227 | 0.975 |
| *Weissella* | 3.365 | 0.636 | 3.553 | 2.157 | 0.934 | 3.016 | 0.731 | 3.204 | 0.66 | 0.849 |
| Average relative abundance of sequences assigned to that genus (mean) constituting 0.4% or more sequences in in each the samples, standard error of the corresponding average (SE) and p-value describing the significance of the differential abundance observed between the two sample sources. Hydroponic-green-treated (HGT); hydroponic-green-untreated (HGU); hydroponic-red-treated (HRT); hydroponic-red-untreated (HRU). | | | | | | | | | | |

**Table S2 (b)**

| Table S1 (b) Bacterial genera (antagonists) in pepper fruit surface samples; between soil untreated and treated green samples, and between soil untreated and treated red samples | | | | | | | | | | |
| --- | --- | --- | --- | --- | --- | --- | --- | --- | --- | --- |
| Genus | SGU mean | SGU SE | SGT mean | SGT SE | p-value | SRU mean | SRU SE | SRT mean | SRT SE | P-value |
| *Acinetobacter* | 6.817 | 0.681 | 7.322 | 0.656 | 0.594 | 4.143 | 0.511 | 4.981 | 0.504 | 0.245 |
| *Agrobacterium* | 0.999 | 0.131 | 1.312 | 0.775 | 0.691 | 0.709 | 0.058 | 0.942 | 0.121 | 0.084 |
| *Arthrobacter* | 1.111 | 0.127 | 1.128 | 0.123 | 0.924 | 1.074 | 0.134 | 1.090 | 0.129 | 0.932 |
| *Bacillus* | 7.199 | 0.674 | 7.280 | 0.626 | 0.929 | 6.299 | 0.571 | 6.381 | 0.524 | 0.916 |
| *Burkholderia* | 7.559 | 1.459 | 8.753 | 1.768 | 0.603 | 4.865 | 0.935 | 6.058 | 1.244 | 0.445 |
| *Curtobacterium* | 3.668 | 0.869 | 4.103 | 1.132 | 0.761 | 2.730 | 0.55 | 3.165 | 0.813 | 0.658 |
| *Enterococcus* | 2.392 | 0.411 | 2.779 | 0.483 | 0.543 | 1.819 | 0.354 | 2.206 | 0.426 | 0.486 |
| *Flavobacterium* | 0.472 | 0.052 | 0.569 | 0.062 | 0.232 | 0.401 | 0.044 | 0.499 | 0.054 | 0.161 |
| *Lactobacillus* | 4.91 | 0.961 | 6.052 | 1.092 | 0.434 | 3.692 | 0.745 | 4.834 | 0.875 | 0.322 |
| *Methylobacterium* | 1.677 | 0.246 | 1.779 | 0.247 | 0.770 | 1.302 | 0.235 | 1.404 | 0.236 | 0.759 |
| *Microbacterium* | 0.871 | 0.162 | 1.019 | 0.191 | 0.555 | 0.699 | 0.128 | 0.847 | 0.157 | 0.466 |
| *Novosphingobium* | 1.057 | 0.159 | 1.149 | 0.166 | 0.689 | 0.633 | 0.099 | 0.889 | 0.141 | 0.216 |
| *Pseudomonas* | 6.323 | 0.872 | 7.484 | 1.151 | 0.423 | 4.267 | 0.537 | 5.428 | 0.815 | 0.236 |
| *Sphingomonas* | 4.154 | 0.969 | 4.164 | 0.969 | 0.994 | 3.158 | 0.611 | 3.169 | 0.610 | 0.989 |
| *Weissella* | 6.433 | 1.116 | 6.621 | 1.045 | 0.902 | 6.084 | 1.141 | 6.272 | 1.069 | 0.904 |
| Average relative abundance of sequences assigned to that genus (mean) constituting 0.4% or more sequences in each of the samples, standard error of the corresponding average (SE) and p-value describing the significance of the differential abundance observed between the two sample sources. Soil-green-treated (SGT); soil-green-untreated (SGU); soil-red-treated (SRT); soil-red-untreated (SRU). | | | | | | | | | | |
